# Tackling a Recurrent Pinealoblastoma

**DOI:** 10.1155/2014/135435

**Published:** 2014-08-25

**Authors:** Siddanna Palled, Sruthi Kalavagunta, Jaipal Beerappa Gowda, Kavita Umesh, Mahalaxmi Aal, Tanvir pasha Chitraduraga Abdul Razack, Veerabhadre Gowda, Lokesh Viswanath

**Affiliations:** ^1^Department of Radiation Oncology, Kidwai Memorial Institute of Oncology (KMIO), Bangalore 560029, India; ^2^Department of Radiology, KMIO, Bangalore 560029, India; ^3^Department of Pathology, KMIO, Bangalore 560029, India; ^4^Department of Anesthesiology, KMIO, Bangalore 560029, India

## Abstract

Pineoblastomas are rare, malignant, pineal region lesions that account for <0.1% of all intracranial tumors and can metastasize along the neuroaxis. Pineoblastomas are more common in children than in adults and adults account for <10% of patients. The management of pinealoblastoma is multimodality approach, surgery followed with radiation and chemotherapy. In view of aggressive nature few centres use high dose chemotherapy with autologus stem cell transplant in newly diagnosed cases but in recurrent setting the literature is very sparse. The present case represents the management of pinealoblastoma in the recurrent setting with reirradiation and adjuvant carmustine chemotherapy wherein the management guidelines are not definitive.

## 1. Introduction

Pineoblastomas are rare, pediatric malignancy of pineal region that account for <0.1% of all intracranial CNS tumors and can metastasize along the neuroaxis with bad prognosis. The present case represents the management of a pinealoblastoma in the recurrent setting.

## 2. Case Presentation

**Figure 1:**
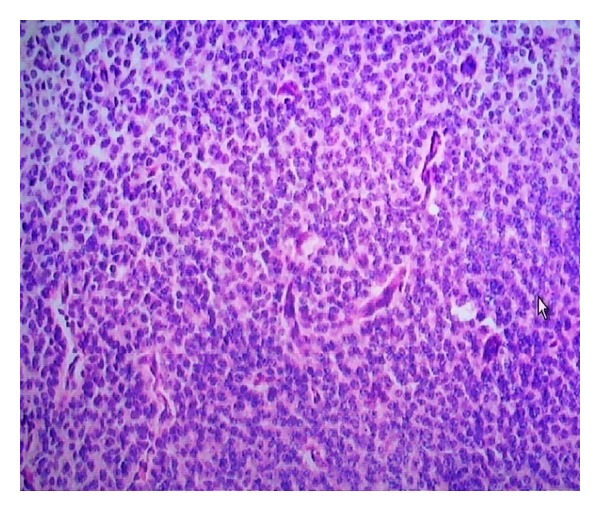
Histopathological slide of pinealoblastoma.

**Figure 2:**
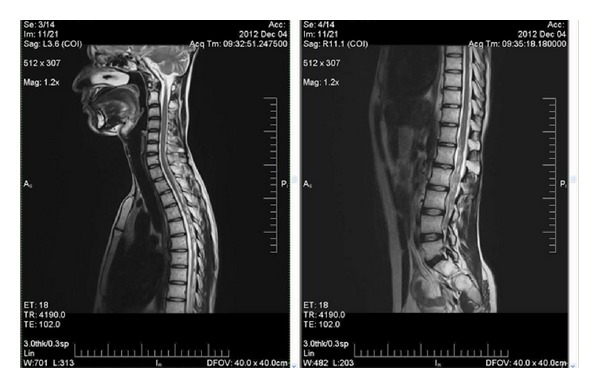
Pretreatment MRI.

**Figure 3:**
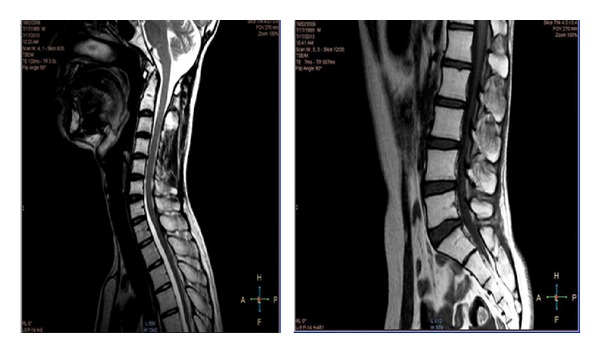
Posttreatment MRI.

A 16 years old boy presented to the hospital with complaints of headache for 1 year, diplopia for 5 days with associated tinnitus, and decreased hearing of the right ear for 5 days. On examination was found to have bilateral (B/L) papilloedema and B/L sixth nerve paresis. A computed tomography scan head revealed a hyperdense mass within the posterior third ventricular region with obstructive hydrocephalus. He underwent a left ventriculoperitoneal shunt and gross total resection of tumour. The histopathology was suggestive of pinealoblastoma (Figure 1). Patient underwent postoperative radiation on telecobalt to the craniospinal axis to a dose of 36 Gy/20 fractions followed with 18 Gy/10 fractions to tumour bed. Patient was on regular followup and was clinically and radiologically NED for 6 years when he developed radiculopathy of 1st lumbar to 1st sacral vertebrae for which an MRI was done which was suggestive of drop metastasis (Figure 2) and a CSF cytology revealed clusters of neoplastic cells. He was treated with reirradiation to the craniospinal axis to a dose of 18 Gy/10 fractions and with carmustine 100 mg/m^2^ intravenously on day 1 and day 2 for 4 cycles in the adjuvant setting. The patient clinically and radiographically (Figure 3) has no evidence of disease at one and half year following reirradiation and single agent carmustine chemotherapy.

## 3. Discussion

Pineoblastomas harbor a poor prognosis and can metastasize along the neuroaxis. Although they typically appear radiographically as a focal enhancing mass, pineoblastomas can be locally invasive and spread outside the pineal region through the subarachnoid space. Pineoblastomas are more common in children than in adults and adults account for <10% of patients [1].

Tumors arising in the pineal area are of germ-cell or neuroectodermal origin. Within the latter category are tumors resembling pineocytes (pineocytoma and less differentiated pineoblastoma), as well as gliomas [2, 3].

Fauchon et al. graded the pineal parenchymal tumours (PPT) into 4 grades based on the mitosis and immunostaining. The present case falls into the Grade 4 category as it corresponds to pinealoblastoma with a high mitotic index and no or weak immunostaining [4].

PPT presents in a fashion similar to germ cell tumors with predominant symptoms related to aqueductal obstruction (raised intracranial pressure) and midbrain compression (Parinaud's syndrome) [5].

Pineoblastomas are managed in a similar fashion to primitive neuroectodermal tumours elsewhere in the CNS. Their prognosis relates, in part, to the same prognostic variables that govern the management of medulloblastoma. Standard-risk patients include those with gross total resections who have no metastases at diagnosis. High-risk patients consist of those with any of the following criteria: minimal resections, positive CSF cytology (M-1), diffuse leptomeningeal metastasis (M-2 or M-3) and diagnosis made when the patient is younger than 3 years.

Infants and children under 3 years of age tend to be treated according to infant brain tumor protocols with intensive chemotherapy alone. Overall, they have a poor prognosis. In the Children's Cancer Group protocol 921, eight infants younger than 2 years of age at diagnosis with pineoblastoma were treated only with the “8 drugs in 1 day” protocol. Under this schedule, all infants developed progressive disease and died. The median Progression-free survival rate was 4 months, and this chemotherapy regimen was judged to be ineffective [6].

For children old enough to receive RT, multimodality therapy (surgery, RT, and chemotherapy) appears to be the preferred method. Chemotherapy may be given before or following RT, as in Children's Cancer Group protocol—local; 36 Gy craniospinal. Any measurable CNS metastases identified during the staging evaluation received additional RT. Results of a relatively large Children's Cancer Group series (15 patients) treated on this randomized protocol are more favorable, with a 3-year progression free survival rate of 61% [6].

In the United States, standard treatment of patients with pineoblastoma currently includes maximal surgical resection followed by adjuvant cranial-spinal irradiation (2520–3855 centigrays [cGy] to the entire axis and 4400–5400 cGy to the tumor site in 200 cGy fractions) and systemic chemotherapy (2 or 3 agents selected from vincristine, cisplatin/carboplatin, cyclophosphamide, etoposide, and carmustine) [7].

Yang et al. in his series of 27 patients of SPNET (supratentorial primitive neuroectodermal tumours) treated with surgery, chemotherapy, and/or radiation had a 3-year progression free survival of 60%, but outcome at 5 years fell to 38% [8].

To overcome the aggressiveness of pinealoblastoma (PB), Sridharan Gururangan et al. in a series of 12 patients used induction chemotherapy, radiotherapy, and high-dose chemotherapy with Autologous stem-cell rescue in children and adults with newly diagnosed pineoblastomas. There were three relapses in local and regional areas. This dictates that the behavior of PB is unpredictable. In the literature there are no specific treatments for these relapsed PB.

Eva Maria Stoiber et al. in their study of long term outcome of adolescent and adult patients with pineal parenchymal tumors treated with fractionated radiotherapy from a single institution had 2 recurrences out of 9 patients. Salvage treatment of the local tumor recurrence consisted of iodine-seed brachytherapy, the spinal metastasis in the lumbar region was reradiated percutaneously (TD 19.8 Gy). 18 months later the patient received chemotherapy (cisplatin and etoposide) due to progressive disease, which was switched to gemcitabine 14 months later.

From NCI cancer data base, there are few studies of recurrent childhood brain tumors wherein they used different combinations of chemotherapies but none has showed good result.

In the present case scenario the patient was treated with craniospinal irradiation postoperatively and was recurrence-free for 6 years when he developed drop metastasis for which he received craniospinal reirradiation and adjuvant carmustine single agent chemotherapy. The patient tolerated treatment well and a good sustained response to therapy was noted.

## 4. Conclusion

This case illustrated the potential benefit of reirradiation and the use of single agent carmustine in the setting of recurrent pinealoblastoma to achieve a good sustained response to therapy. Because of the rarity of pineal tumors, management at recurrence does not exist and further studies are needed to have well defined protocols in the setting of recurrent pineal tumours.
